# Stress and eating behaviors in young subjects can influence early cardiovascular prevention

**DOI:** 10.1590/0102-311XEN017424

**Published:** 2024-03-11

**Authors:** Anna Vittoria Mattioli

**Affiliations:** 1 University of Bologna, Bologna, Italy.

Dear Editors,

We have read with great interest the article *Adherence to the Mediterranean Diet and Depression, Anxiety, and Stress Symptoms in Chilean University Students: A Cross-Sectional Study* by Morales et al. ^1^, and consider it significant in the field of early cardiovascular prevention.

The authors found that adherence to the Mediterranean diet and consumption of fruit, vegetables, nuts, avocado, fish, and seafood are associated with a lower likelihood of depression in Chilean university students.

Regarding the findings reported in the paper, we would like to make the following contribution to the discussion. Atherosclerosis - which is the basis of cardiovascular diseases - is a long process that begins in the childhood and is the main cause of cardiovascular events in adulthood and old age ^2^ ^,^ ^3^. Unfortunately, it is known that there is a lack of awareness on the relevance of early intervention, especially among young people and women ^4^ ^,^ ^5^. Stress and depression exert a notable influence on dietary behaviors, manifesting in various ways that differ among individuals. The relation between stress and diet is complex, and its effects can be both immediate or long-lasting. Common patterns are emotional eating and cravings ^6^ ^,^ ^7^ ^,^ ^8^. Emotional eating is a coping mechanism that often involves consuming foods that provide comfort, with high content of calories, sugar, or unhealthy fats. Furthermore, stress can induce specific cravings, typically for sugar-, fat-, and salt-rich foods ^6^ ^,^ ^7^ ^,^ ^8^. Under stress, individuals may opt for convenience foods that are quick to obtain but often lack nutritional value, contributing to unhealthy eating habits. Stress can also disrupt normal eating habits, leading to either overeating or undereating. Both patterns can hold implications for overall health ^7,8^. Obesity induces an inflammatory state characterized by an increase in leptin levels, a pro-inflammatory adipokine, and decreased levels of adiponectin, an anti-inflammatory adipokine, have been reported ^7^ ^,^ ^8^ ^,^ ^9^ ^,^ ^10^. Inflammation has been strongly associated with the development of atherosclerosis. Regular physical activity helps in the fight against obesity and inflammation. An additional beneficial effect of regular physical activity in managing stress has been suggested ^11^. The Mediterranean diet offers many benefits for health ^12^ ^,^ ^13^. Undoubtedly, fruit and vegetable intake is a good index for assessing a good diet and, as is known, it is extremely variable in young people and also shows differences between sexes ^14^ ^,^ ^15^ ^,^ ^16^. Ensuring an adequate intake of vitamins and incorporating a variety of fruit and vegetables into the diet is crucial for the overall health of young individuals and can contribute to the prevention of cardiovascular disease by various mechanisms ^17^.

Young subjects are less likely to adopt a healthy lifestyle ^18^ ^,^ ^19^. Ünal & Özenoğlu ^20^ reported that adherence to the Mediterranean diet was decreasing among university students. Most students (59.2%) had poor Mediterranean diet adherence. Compared to poor adherers, subjects with good adherence to the Mediterranean diet had significantly lower depression scores and stress levels in both males and females. These results confirmed what was reported by Morales et al. ^1^.

To prevent the early development of atherosclerosis with a notable benefit in the onset of cardiovascular disease with age, it is important to raise young people’s awareness of the importance of a proper lifestyle. The recent decline in lifestyle in the *Life’s Essential 8* and the inherent connection with stress and depression facilitate the identification of areas in which action should be taken ^21^ ^,^ ^22^ ^,^ ^23^. Tailored interventions for young people are needed.

How to intercept young people is the key question: for young women, pregnancy seems to be an optimal time to assess cardiovascular health and promote the adoption of virtuous behaviors ^24^ ^,^ ^25^.
